# Laparoscopic appendectomy for complicated appendicitis in children: does the post-operative peritoneal drain make any difference? A pilot prospective randomised controlled trial

**DOI:** 10.1007/s00383-022-05155-6

**Published:** 2022-06-30

**Authors:** M. J. Human, N. Tshifularo, M. Mabitsela

**Affiliations:** Department of Paediatric Surgery, Sefako Makgatho Health Sciences University, Dr George Mukhari Academic Hospital, Molotlegi Road, Ga-Rankuwa, Pretoria, 0208 South Africa

**Keywords:** Laparoscopy, Complicated appendicitis, Peritoneal drain, Randomised, Prospective

## Abstract

**Purpose:**

This was a pilot randomised, prospective study, which aimed to determine and compare the post-operative complications of paediatric patients undergoing laparoscopic appendectomy (LA) for complicated appendicitis, with and without a peritoneal drain.

**Methods:**

Patients younger than 13 years, undergoing LA for complicated appendicitis at the Dr George Mukhari Academic Hospital (DGMAH), over a 15-month period during 2019–2020 were enrolled. Randomisation was achieved by a blocked randomisation plan. Patients were randomised in a 1:1 ratio into the “drain” (D) and “no drain” (ND) groups.

**Results:**

Thirty-four patients were included in this study; seventeen in each group. The complication rate was 26%. Intra-abdominal collection accounted for 89% of the complications. The complication rate in the “D” group was 18% and 35% in the “ND” group, with no statistically significant difference. Complication rates were higher (38%) in patients with generalised pus when compared to localised pus (7%), although not statistically significant. The mean theatre time, hospital stay, and duration of antibiotic use did not differ significantly between the groups.

**Conclusion:**

From our study, the post-operative peritoneal drain did not make any statistically significant difference in patient outcome. The amount of intra-abdominal contamination is more likely to contribute in the development of complications.

**Trial registration number:**

SMUREC/M/15/2019: PG.

## Introduction

Acute appendicitis is a common pathology encountered by paediatric surgeons. Laparoscopic appendectomy (LA) has been established as a safe treatment modality and is the treatment of choice for simple appendicitis [1–3]. Laparoscopy offers the advantages of magnified visualisation of operating field, shorter hospital stays, improved cosmesis, and fewer wound-related complications [1, 3–5].

Complicated appendicitis refers to a perforated/gangrenous appendix with or without abscess formation [6]. Most of our patients present with complicated appendicitis due to delayed diagnosis, transport difficulties, and delayed health-seeking behaviour. The rationale in post-operative drain intra-abdominal placement is draining remaining infective fluid and thus in theory, limiting the post-operative IAC. The need for prophylactic post-operative drain placement is increasingly being questioned.

Limited studies address the question of “drain or no drain” in children, with most available studies being retrospective. This was a first-of-its-kind study in the country, which addresses this important question, since most of our patients present with complicated disease. This was a randomised, prospective study, determining and comparing the post-operative complications of paediatric patients undergoing LA for complicated appendicitis, with and without a peritoneal drain.

## Methods

### Study setting

This pilot study was conducted in the Paediatric Surgical Ward in DGMAH, a tertiary hospital situated in Ga-Rankuwa, Gauteng, South Africa. The study was conducted over a 15-month period during 2019/2020.

### Study design and randomisation plan

This is a quantitative, comparative, randomised prospective study of children undergoing LA for complicated appendicitis at DGMAH. Patients were randomly and equally divided into the “drain” and “no drain” groups as they presented to the Paediatric Surgical Ward (Ward 18). Randomisation was achieved by a blocked randomisation plan. Patients were randomised in a 1:1 ratio into the “drain” (D) and “no drain” (ND) groups. Children younger than 13 years undergoing LA for complicated appendicitis at the DGMAH for the period 2019–2020 were enrolled in the study. The age cut-off is based on the hospital cut-off for paediatric patients. There were no changes in the methods after the initiation of the trial.

### Study population, sample, randomisation plan, and data collection

Following ethics approval from Sefako Makgatho Health Science University ethics committee (SMUREC) and trial registration, we conducted a randomised prospective study, which was the first of its kind in the Department of Paediatric Surgery at Sefako Makgatho Health Science University. It generated valuable information on the occurrence of post-operative complications following LA for complicated appendicitis in children with and without a peritoneal drain. It is estimated that approximately 45 patients meeting the inclusion criteria could be recruited in 15 months of data collection. This figure is based on an average admission rate of approximately 3 patients per month. Patients were be randomised in a 1:1 ratio into a “drain” or “no drain” group. However, our sample size was smaller due to lower patient numbers. Five patients that met the criteria chose not to participate and were excluded. In four patients that met the criteria for the study, the surgeons did not follow the randomisation plan correctly and were excluded. 34 were recruited in the period of data collection. Patients were randomised in a 1:1 ratio into a “drain” or “no drain” group, namely 17 patients per group.

Patients were admitted in the Paediatric Surgical Ward in DGMAH by the surgeon on duty with a suspected acute appendicitis. Written consent was obtained from the guardian in all potential suitable patients. Guardians were allowed to refuse or withdraw from the study at any time. Children over the age of 7 years were allowed to sign assent together with their guardians. A blocked randomisation plan whereby patients were randomised on a 1:1 ratio into “drain” and “no drain groups” was used.

Once complicated appendicitis had been confirmed by the operating surgeon with intraoperative laparoscopic findings, the decision to leave a drain or not was dictated by the blocked randomisation plan, which was available in the surgical ward and theatre. All patients received standardised antibiotics: Ceftriaxone (50 mg/kg BD) and Metronidazole 15 g/kg TDS). The duration was dependant on inflammatory markers on day 3 and 5 post-operative of antibiotics and the clinical parameters of the patient. Theatre duration or time was defined from induction of anaesthesia. The standard operative technique was used in both groups, consisting of an open Hasson technique for port insertion. Three port techniques were used, one camera port and two working ports. A 14 mm open pencil drain was used in the “drain’’ group. The drain would be placed in the suprapubic port position. All drains were left in situ for minimum of 24 h. The drain would be removed based on the drainage from the drain. Criteria for removal included no draining of puss for more than 24 h or only serous fluid draining. Suction of pus without irrigation was standard in both groups. A distinction between localised and generalised puss was made where generalised puss was defined as puss in all four quadrants of the peritoneal cavity. A questionnaire was completed by the operating surgeon during the post-operative period. Patients were followed up in the Outpatient Department 2 weeks post-hospital discharge or contacted telephonically if they were unable to visit the Outpatient Department. Data were extracted from the questionnaires and entered onto a Microsoft Excel database on the principal investigator’s laptop.

The data sheet included, amongst other data, the following: patient demographics, procedure-related data, and post-operative data (duration of antibiotic use, complications, and duration of post-operative hospital stay). Demographic and clinical characteristics of patients were summarised descriptively. No changes of outcomes were instituted after the initiation of the trial. Continuous data were summarised by mean, standard deviation, median interquartile range, and minimum and maximum values. Categorical data were summarised by frequency counts and percentage calculation.

### Data analysis

The rate (percentage) of post-operative collection was calculated for patients in each group (“drain”/’no drain”) by Fisher’s exact test. A similar comparative analysis was performed for the post-operative surgical site infection, paralytic ileus, and other post-operative complications. The mean length of hospital stay was compared between the two groups using the two-sample t test. The median hospital stay was compared using the nonparametric Wilcoxon rank sum test.

All statistical analyses were performed on SAS (SAS Institute Inc., Carey, NC, USA), Release 9.4 or higher, running under Microsoft Windows for a personal computer. Statistical tests are two-sided and *p* values ≤ 0.05 (5%) were considered significant.

No additional or subgroup analysis was performed.

## Results

Of the patients meeting the inclusion criteria, 34 were recruited in the period of data collection. Patients were randomised in a 1:1 ratio into a “drain” or “no drain” group, namely 17 patients per group. The study population (56%) consisted of males. African race comprised the majority (94.1%) of the study population. The mean age was 9.2 years in the study group. The difference in gender, race, and age distribution was not statistically significant (Table 1). The mean theatre time, duration of hospital stay, and antibiotic use did not differ significantly between the two groups (Tables 2, 3, 4). Intraoperative generalised pus was encountered in 62% (21/34) of patients. The complication rate in the study group was 26% (9/34). Intra-abdominal collection accounted for 89% (8/9) of the complications. Complication rates were noted to be higher (38%) in patients with generalised pus when compared to localised pus (8%); however, this was not statistically significant (Table 5). The complication rate in the “D” group was 18% and 35% in the “ND” group (Table 6). The complication rate between the two groups did not differ significantly. Relook laparoscopy was performed in 67% (6/9). One patient was treated conservatively with antibiotics for IAC. An image-guided pigtail drain insertion was used in one patient with IAC. A relook laparotomy was required in one patient that sustained a bladder injury during port insertion and developed IAC.

**Table 1:** Demographic characteristics

Characteristic	No drain (ND) (*n* = 17)	Drain (D) (*n* = 17)	*p* Value
Gender			
Male	11 (64.7%)	8 (47.1%)	0.491*
Female	6 (35.3%)	9 (52.9%)	
Race			
African	17 (100%)	16 (94.1%)	1.000*
Mixed race	–	1 (5.9%)	
Age (years)			
Mean (± SD)	9.2 (± 2.46)	9.2 (± 2.21)	0.942**
Median (IQR)	10 (8–11)	9 (8–11)	0.862***
Minimum/maximum	4/12	5/12	

*Fisher exact test

**Two-sample *t* test

***Nonparametric Wilcoxon rank sum test

**Table 2:** Theatre time

Theatre time (h)	No drain (*n* = 17)	Drain (*n* = 17)	*p* Value
Mean (± SD)	2.32 (± 0.68)	2.44 (± 1.02)	0.696*
Median (IQR)	2.3 (2.0–2.5)	2.2 (1.8–2.7)	0.972**
Minimum/maximum	1.1/3.8	1.5/5.8	

*Two-sample *t* test

**Nonparametric Wilcoxon rank sum test

**Table 3:** Duration of antibiotic treatment

Duration of treatment (days)	No drain (ND) (*n* = 17)	Drain (D) (*n* = 17)	*p* Value
Mean (± SD)	6.2 (± 2.63)	5.7 (± 2.57)	0.557*
Median (IQR)	6 (4–8)	5 (5–6)	0.484**
Minimum/maximum	3/13	3/14	

*Two-sample *t* test

**Nonparametric Wilcoxon rank sum test

**Table 4:** Hospital stay

Hospital stay (days)	No drain (*n* = 17)	Drain (*n* = 17)	*p* Value
Mean (± SD)	6.24 (± 2.68)	6.24 (± 2.36)	1.000*
Median (IQR)	6 (4–8)	5 (5–7)	0.930**
Minimum/maximum	2/13	4/14	

*Two-sample *t* test

**Nonparametric Wilcoxon rank sum test

**Table 5:** Complications (generalised pus vs localised pus)

Complications	Generalised pus (*n* = 8)	Localised pus (*n* = 1)	*p* Value
Collection	6 (75.0%)	1 (100%)	
Collection, bladder perforation	1 (12.5%)	–	1.000*
Wound sepsis	1 (12.5%)	–	

*Fisher exact test

**Table 6:** Complications by drain

Complications	No drain (*n* = 6)	Drain (*n* = 3)	*p* Value
Collection	5 (83.3%)	2 (66.7%)	
Collection, bladder perforation	–	1 (33.3%)	0.583*
Wound sepsis	1 (16.7%)	–	

*Fisher exact test

## Discussion and conclusion

Acute appendicitis is a common surgical emergency encountered by paediatric surgeons, with an increase in incidence with age. The lifetime risk of developing acute appendicitis is around 7% [7]. In the infantile group, it is 1 in 10,000 to approximately 20 per 10,000 in children under 14 years [8]. Less than 5% of appendicitis cases get diagnosed before the age of 5 years [9]. Our average age was lower at 9 years. There was a male (56%) predominance over females in our study group, which is in keeping with international data [8]. There was no statistical significance in the gender and age distribution in the “D” and “ND” groups. Most of our patients were of African race, which is keeping with the race distribution of our hospital drainage area. There was no statistical difference in the race distribution between the “D” and “ND” groups. Even though acute appendicitis is one of the most encountered surgical pathologies, many of the management aspects remain controversial, from simple to complicated appendicitis.

Appendicitis has a diverse clinical presentation from simple to complicated appendicitis with septic shock. A delay in the correct diagnosis is the reason that pre-schooler with appendicitis present with more complicated appendicitis when compared to their older counter parts [9, 10]. Patient geography and socioeconomics also play an important role, especially in our setting, where availability of patient transport is limited and often far away from a centre that offers paediatric surgical services. All the above factors contribute to a delay in health-seeking, accurate diagnosis, and appropriate treatment. As a result, over 80% of our patients present with complicated appendicitis, which is significantly higher than the international figure of 30% [11].

In complicated appendicitis, various controversies and debates surround the optimal operative technique. First is the definition of complicated appendicitis. Most surgeons agree that an appendicitis associated with a perforation, faecolith, gangrene, or abscess is complicated [6].

Complicated appendicitis is associated with higher rates of post-operative complications. The most common complications include IAC, surgical site infection, and prolonged post-operative ileus. More than 80% of our patients present with complicated appendicitis, of which 91% are managed with LA. This is contrast with developed countries’ data where most patients present with simple appendicitis. In our practise, laparotomy is reserved for patients who are severely ill, hemodynamically unstable and often in septic shock. With post-operative IAC being one the most common complications, various controversial strategies exist to limiting this complication. The most common strategies include peritoneal irrigation and post-operative peritoneal drain.

The use of peritoneal irrigation stems from an old principle of “dilution is the solution to pollution”. This, however, has been challenged by St Peter et al. and a more recent meta-analysis, showing no benefit in preventing post-operative IAC in adults and children [12, 13]. In a randomised prospective study conducted at our institution on adults, potential harm was indicated with irrigation [14]. Based on this evidence, “suction only” was performed in all the patients in the study group.

The use of surgical drains dates to Hippocrates (460–377 BC) for the treatment of empyema [15]. The rationale for draining residual fluid is that it decreases the volume of infected fluid, thereby decreasing the probability of a post-operative collection formation. Post-operative peritoneal drain placement is still frequently used for various surgical pathologies which are institution- and surgeon-dependent. Its role in complicated appendicitis is being challenged. There are limited international randomised prospective data supporting the use of drains, even more so in children. During the nineteenth century, it was highlighted that drains might have associated complications. There are no prospective studies supporting the use of post-operative peritoneal drains. Multiple studies have since then highlighted these complications including surgical site infections, longer hospital stay, longer antibiotic, and analgesia use [16–19]. Therefore, the data suggest no difference in outcome, but a potential risk associated with the use of peritoneal drains.

From this study, it appears that the drain did not make a statistical difference in patient outcomes. The data show that we have a complication rate of 26% of patients undergoing LA for complicated appendicitis. Of our complications, 89% were intra-abdominal collections, where 66% of them required reoperations. Four (4) relook laparoscopies as well as 1 relook laparotomy were done. One (1) intra-abdominal collection was treated conservatively with culture-directed antibiotics. Two-thirds of the complications arose from the “ND” group. The incidence of complications in the “ND” group was 35% compared to 18% in the “D” group, which was not statistically significant. This difference could be of clinical significance; however, larger study groups is required to draw a conclusion. Theatre time was prolonged in the “D’’ group, which was most likely due to the additional procedural step of inserting a peritoneal drain.

It is evident that the amount of contamination plays a more important role in determining the probability of developing complications, as 38% of patients with generalised pus developed complications compared to 7% of patients with localised pus. It, however, did not reach statistical significance, but could indicate a clinical significance.

## Conclusion

The use of the post-operative peritoneal drain in the management of complicated appendicitis is increasingly being questioned with no prospective studies showing any benefit. It is clear from our study that the peritoneal drain did not have a statistically significant difference in patient outcomes. Post-operative complications are most likely multifactorial and related to the severity of contamination. We acknowledge that this is a single institution with a small study group with limited ability to draw a firm conclusion.

## Abbreviations

LA: Laparoscopic appendectomy
DGMAH: Dr George Mukhari Academic Hospital
D: Drain
ND: No drain
IAC: Intra-abdominal collection
